# Doubt About Prediction Role of S100B Protein in Brain Death

**DOI:** 10.5812/atr.17351

**Published:** 2014-06-20

**Authors:** Ali Kabir, Mehrdad Moghimi, Afshin Amini

**Affiliations:** 1 Minimally Invasive Surgery Research Center, Iran University of Medical Sciences, Tehran, IR Iran; 2 Department of Epidemiology, Faculty of Public Health, Shahid Beheshti University of Medical Sciences, Tehran, IR Iran; 3 Department of Surgery, Shahid Beheshti University of Medical Sciences, Tehran, IR Iran; 4 Department of Toxicology and Emergency Medicine, Faculty of Medicine, Imam Hossein Hospital, Shahid Beheshti University of Medical Sciences, Tehran, IR Iran

**Keywords:** Brain Death, S100b Protein, Human, Control Groups, Decision Support Techniques

Dear Editor,

We read with interest the recent paper about S100B protein as a biomarker for prediction of brain death by Shakeri et al. (1). There are some notes, which may help in more clarification of the paper. To answer the important question raised by this study, we need two groups: one group with brain death and the other one without brain death. Our findings should be compared between these two groups.

 However, according to explanations given by the authors, we believe that they have confused the meaning of the main questions and have compared the results of the survivors with dead cases and brain death cases according to their explanation in Table 3. Authors have mentioned that “The correlation between pupillary response and S100B protein level at different stages was not significant.” If they have analyzed data of each stage according to sampling for S100B protein, they would have found the low and insufficient sample size in each subgroup which dictates no significant association.

 In such occasions, we need to know the considered power of this statistical test for evaluation of the association. Surely, with each low power we will reach to nonsignificant association, which is useless. As time passes, the power of association between GCS (first or final) and S100B protein increases as has been shown by values of Pearson correlation coefficient. This is a matter that can be considered in predicting the value of S100B protein and GCS instead of each other, specifically between primary GCS and final S100B level.

 When we are unable to measure S100B, or we want to calculate it earlier in the course, we can use it by the coefficient of determination equal to 0.85 (which is determined by square of the correlation coefficient; here it is calculated as 0.92^ 2^). Despite the fact that our focus is on using S100B protein as a biomarker for predicting brain death; however, estimating the final value of S100B at the beginning of the study according to the GCS status is highly valuable; an issue which has been ignored by authors.

 Authors have tried to use S100B protein as a post-traumatic biomarker for the prediction of brain death; nevertheless, it has been determined after brain death in some cases as authors have mentioned it in their methods. In prediction models, we use variables, which occur before outcome. Changes in S100B protein after brain death make it useless to determine the predictive power of this marker. It is not clear that what authors mean by last GCS? Is this pertaining to last existing GCS, before brain death or other time?

 The same issue should also be considered for measurement of final S100B protein. Such measurements are not reproducible in future studies. We are not aware of the exact time of these measurements. How is it feasible that all selected patients be examined on admission, discharge or death by one resident who is blind about the selection of the patients? Has he worked up all other patients or was there more than one assessor? How many patients are admitted to your ward? How many neurosurgery residents do you have in your hospital? Do they have sufficient agreement with each other in clinical assessment?

 In Table 2, it is not defined that what these values are pertaining to? Which times or which patients or different grades of CT scan are they? However, it is not important that which one it is because all cases have been considered at once. We should compare cases with brain death with others (dead or alive cases). Such kind of comparison is conclusive. So, we cannot conclude that only S100B protein after 48 hours can predict brain death, major conclusion by the present study. In Table 3, it is not clear whether authors have compared three or four groups with each other?

For exaggerating statistical difference between subgroups, we are highly prohibited from such two by two comparisons between subgroups. It will induce multiple comparison biases. Instead, we can use post hoc tests.

## References

[1] Shakeri M, Mahdkhah A, Panahi F (2013). S100B Protein as a Post-traumatic Biomarker for Prediction of Brain Death in Association With Patient Outcomes.. Arch Trauma Res..

